# The Role of the Human Mirror Neuron System in Supporting Communication in a Digital World

**DOI:** 10.3389/fpsyg.2017.00698

**Published:** 2017-05-12

**Authors:** Kelly Dickerson, Peter Gerhardstein, Alecia Moser

**Affiliations:** ^1^U.S. Army Research Laboratory, Human Research and Engineering, AberdeenMD, USA; ^2^Department of Psychology, Binghamton University, BinghamtonNY, USA

**Keywords:** mirror neurons, imitation, screen-based communication, correspondence problem, exaptation, embodied cognition

## Abstract

Humans use both verbal and non-verbal communication to interact with others and their environment and increasingly these interactions are occurring in a digital medium. Whether live or digital, learning to communicate requires overcoming the correspondence problem: There is no direct mapping, or correspondence between perceived and self-produced signals. Reconciliation of the differences between perceived and produced actions, including linguistic actions, is difficult and requires integration across multiple modalities and neuro-cognitive networks. Recent work on the neural substrates of social learning suggests that there may be a common mechanism underlying the perception-production cycle for verbal and non-verbal communication. The purpose of this paper is to review evidence supporting the link between verbal and non-verbal communications, and to extend the hMNS literature by proposing that recent advances in communication technology, which at times have had deleterious effects on behavioral and perceptual performance, may disrupt the success of the hMNS in supporting social interactions because these technologies are virtual and spatiotemporal distributed nature.

## Introduction

Social interactions are complex and dynamic exchanges, typically involving both verbal and non-verbal components. These interactions are clearly adaptive, supporting group and individual success. Social interactions are communicative, and can convey information about the environment (Want and Harris, 2002) or the cultural context (Gergely and Csibra, 2006; Liszkowski et al., 2008). For example, both children and chimpanzees use social transmission to learn about tool use (Nagell et al., 1993; Flynn and Whiten, 2008). In a similar vein, facial expressions and postural changes are used to communicate changes in emotional states (Avenanti and Aglioti, 2006; Freedberg and Gallese, 2007). Social communication (both gesture and language) can be used to demonstrate and transmit cultural traditions in human groups, but also in non-human primates (Perry and Manson, 2003). All of these complex interactions rely on the ability of an observer to extract meaning from actions produced by others. Extracting meaningful information about the environment or a cultural exchange by watching or participating in an exchange with another is broadly defined as observational learning (Tomasello et al., 1987). This type of learning is computationally and cognitively demanding, and requires solving a correspondence problem: Resolving the apparent mismatch between cues available to the sensory system of an observer and their source, the motor system (and the underlying intentions) of an actor attempting to convey meaningful information (see Mitchell, 2011 for the Theory of Mind perspective).

Several solutions to the correspondence problem have been proposed. Notable examples include Meltzoff’s (2007) ‘like me’ framework, which suggested that early in development infants begin linking the actions of others to their own actions (and vice versa). The ‘like me’ framework posits that the correspondence problem is resolved early in life by representing others in the context of the self. Importantly, these representations are generated based on internal sensorimotor transformations that occur during action observation, and not based on the generation of the observer’s own motor output. Hasson et al. (2012) brain-to-brain coupling account offers a similar description: Social interactions are characterized by the exchange of signals (e.g., speech, touch, gestures) emitted by an individual and transmitted across a shared environmental context. Social interactions and the associated signal exchange follow social rules and norms, which provide an underlying statistical structure that the brain can harness and interpret. Brain-to-brain coupling has been suggested as a mechanism supporting joint behaviors such as verbal and non-verbal communication, imitation, and other forms of social learning (e.g., emulation and mimicry; see Want and Harris, 2002). Brain-brain coupling has been proposed to account for action understanding by mapping meaning onto arbitrary gestures, and to support speech perception and production by linking or *binding* the sensory and perceptual systems of an observer to the motor system of an actor. This perceptual-motor *coupling* enables coordinated and dynamic perception-production cycles both within and across individuals, which support complex and meaningful social interactions through gesture and language (Hasson et al., 2012).

The idea that a dyad can coordinate their activity through a shared signal is certainly not new (Piaget, 1951; Meltzoff and Borton, 1979; Tomasello, 1996; Meltzoff, 2007). Accounts characterizing observational learning have provided only descriptions of how one might solve the correspondence problem, with little discussion of the underlying mechanisms that might support a solution. There is ample evidence, however, that gestural and linguistic communication is supported by a single overarching system, the human mirror neuron system (hMNS). This system evolved to support a link between perception and action in both gestural and linguistic domains (Rizzolatti and Arbib, 1998). The mirror neuron system (MNS) contains a class of neurons that respond to both observed and self-produced actions (Di Pellegrino et al., 1992; Rizzolatti and Arbib, 1998). Mirror neurons are thought to support observational learning and promote imitation (Cross et al., 2009). In humans, mirror neurons are located in a part of the brain that is predominantly involved in speech perception and production. This has lead many to suggest that hMNS reflects exaptation, the evolutionary “repurposing” of a system of gestural communication, to support both verbal and non-verbal communication and interaction (Arbib, 2005, 2010). In line with this argument is Rizzolatti’s (2005) assertion that the hMNS likely underlies a range of social and communicative functions, all of which share in common the need to translate “pictorial descriptions of observed actions” from higher order visual representations to motor representations.

The information processing and sensory transformations required to solve the correspondence problem and learn from the gestures and expressions of those around us are demanding even under the most ideal conditions of real-time face-to-face interactions. The use of digital interfaces for communication and learning introduces additional potential difficulty in translating visual information to action. Currently, the impact of moving an interaction from real-time to a partial or fully virtual space on perception and action understanding is not well understood. The research literature has effectively documented many of the parameters of dyadic interactions in the real world, but the availability of new communication technologies has outpaced the available research. The emergence and broad acceptance of virtual and distributed communication has presented a unique opportunity to re-examine the neural and behavioral systems that support resolution of the correspondence problem and enable social interactions.

### Learning from Digital Interactions across the Lifespan

2D communication technology has advanced considerably beyond video presentations, in both perceptual realism and physical immersion, and these advances have decreased the differences between virtual and natural learning environments. Despite these technological achievements, recent research with infants and adults suggests that differences in information processing and learning outcomes persist. For example, young children demonstrate poorer learning from screen media sources (See video deficit effect, Anderson and Pempek, 2005; Zack et al., 2009; Krcmar, 2010) and adults demonstrate poor emotional fluency while using video based communication systems (Wallbott, 1992; Kappas and Krämer, 2011). Given the converging evidence across learning environments and developmental periods, we propose that the exaptation of the neural architecture supporting communication (hMNS) in the real world operates less efficiently and effectively in the presence of temporal, spatial, and social disruptions inherent to virtual communication and screen media platforms. This assertion is based on an emerging literature demonstrating that the temporal and spatial decoupling in virtual- and screen-based interactions are fundamentally disruptive to processing communicative information, both gestural and linguistic.

### Differences in Learning between 2D and 3D in Adults and Children

Generally, the developmental literature on learning from and interacting with screen media is more extensive than that testing adults. The performance of children in a digital media context enables insights into the extent to which a developing MNS responds to changes in cues that reduce the face-to-face nature of an interaction. Behaviorally, young children demonstrate poorer learning from screen media compared to live interactions (see DeLoache et al., 1998; Barr, 2010; and Dickerson et al., 2013 for descriptions of the *video deficit effect*). Evidence of differential neural processing of video compared to live social demonstrations in infants (Shimada and Hiraki, 2006), toddlers (Ruysschaert et al., 2013), and children (Moriguchi and Hiraki, 2014) are consistent with research on the video deficit. Shimada and Hiraki (2006), for example, found that 6- to 7-month-olds observing live motor demonstrations involving objects had greater sensorimotor activation (measured via NIRS) during an observation that involved an actor manipulating the object compared to observation of the object moving independently of the demonstrator (i.e., object reenactment, “ghost” condition). Further, when they compared these effects between live and video-based demonstrations, the enhancement provided by the social agent was not evident in the video condition, leading the authors to suggest that video learning may not evoke the same mirroring responses of actions between self and other. Parallel findings with toddlers have emerged in the literature as well; in a study with 18- to 36-month-olds performing a goal-directed imitation task, Ruysschaert et al. (2013) found that mu suppression, a measure of neural mirroring, during imitation of demonstrated actions was greater following live than video demonstrations. Together, it appears that MNS processing differences between live and video demonstrations is characteristic of early development.

While the behavioral gap in learning outcomes between live and video learning may be closing in older children, differences in engagement of the hMNS persist (Moriguchi and Hiraki, 2014). Moriguchi and Hiraki (2014) investigated live and TV learning in children ages 5–6 years using NIRS. Children played a matching card game, and despite showing similar patterns of behavioral results between live and TV learning conditions, there was marginally less (with a moderate effect size) recruitment of the left primary motor cortex and significantly more occipital activation during observation of TV versus live demonstration. Importantly, these patterns are consistent with those of adults, who have been found to exhibit weaker activation in the primary motor cortex during gesture observation in video compared to live conditions across several imaging techniques (Järveläinen et al., 2001). More visual processing of the 2D stimulus has also been observed in adults, again demonstrating parallel patterns between children and adults (Perani et al., 2001; Carver et al., 2006).

These examples suggest that virtual (video) social interactions provide cues that are significantly different from the cues available during live interactions, and appear, to a certain extent, to mediate behavioral learning outcomes. While the observed behavioral disruptions may be resolvable, depending on age and task demands, changes in social information continue to challenge information processing at a cortical level. The type of low-level social cues that could disrupt hMNS or other regions of processing could be as subtle as changes in gaze direction (Demers et al., 2013), body posture, and speech cadence (see also Saarni and Harris, 1991). Further, perceptual and social contingency cues differ and are potentially degraded in screen media and virtual interactions, which minimally would lead to an increase in the resources required to learn from the degraded content (Moser et al., 2015). For example, color and brightness cues are often different, the size and space occupied by the actor of the screen is unrealistic, and, most salient, the resolution of the information is usually substantially lower than during a live interaction. Further, “interactive” children’s programs (e.g., Blue’s Clues and Dora the Explorer) that attempt to engage the child by encouraging participation in joint activities (e.g., counting) and verbal responses to questions are generally non-contingent; that is, actors and observers are spatially and temporally decoupled and the actions of one cannot influence the actions of the other to the same extent as closed-circuit television (CCTV; e.g., a video-chat context) or face-to-face interactions. It is generally accepted that both temporal and spatial contingencies are important for social interactions (see Troseth et al., 2006; Anderson and Hanson, 2010; Dickerson et al., 2013). For instance, increased visual attention to task goals has been captured in a CCTV setting and these differences were indicative of better performance on the task (Taylor and Herbert, 2014). Changes in perceptual cues and social contingency contribute to children’s difficulty in learning from screen-based media, even when it is intended to be social and interactive. Social scaffolding from live (Zack et al., 2013; Zimmermann et al., 2016) and CCTV (Troseth et al., 2006; Taylor and Herbert, 2014) interactions have been found to produce substantial improvements in learning outcomes.

## Realism and Immersion: Virtual and Mixed Reality Social Interactions

Many of the examples cited so far deal with comparisons between live and video-based interactions. However, new technologies are enabling increasing levels of realism and immersion, where learners are no longer just passively viewing a demonstration presented in 2D, but rather are engaged with an interactive 2D or constructed 3D display and interacting with either real or virtual objects (mixed reality, MR, and virtual reality, VR, respectively). Changes in immersion and realism, however, are often implemented at the feature level (e.g., biological motion, see Beauchamp et al., 2003 for example), and may not lead to meaningful improvements in perceived contingency within an interaction or in critical spatial and temporal parameters.

One of the earliest studies (Perani et al., 2001) to take on this issue used PET and included four observation conditions (Reality, Virtual Reality (VR) high realism, VR low realism, TV). Activation of the right inferior parietal cortex was exclusive to the reality condition, suggesting that only actions executed in real 3D engaged areas in the brain associated with visuospatial information supporting action representations. Actions executed in VR, both with high and low realism and over TV, produced activation in predominantly lateral and mesial occipital regions, which are involved in supporting object perception but have not been found to support action representations. A later EMG study (Electromyography, measures activity of primary motor cortex), was used to quantify differences in muscle activity of an observer during the demonstration of a to-be-imitated task via human over video, robotic, or android demonstrator. The robotic demonstrator differed from the human in form and motion. The android differed in motion, such that it had a likeness in form to the human, but the motion of the robot. Hofree et al. (2015) observed a similar pattern of behavioral results across the three different task demonstrations, but EMG responses showed greater synchronization in human compared to other conditions across both observation and imitation trials. The authors suggest that this difference could be explained by the MNS being specialized to mirror biological agents (Miller and Saygin, 2013), or potentially more simply, a sensitivity to “temporal fidelity” of action observation and execution (Hofree et al., 2015).

Temporal fidelity has been discussed in evaluating performance in other technologies as well. For example, Parkinson and Lea (2011) found that disruptions in emotion processing are likely the result of the temporal asynchronies inherent in web-based video conferencing (see Manstead et al., 2011 for review). One possible approach to addressing the limitations of fully virtual or digital information transmission is to use tactile virtual reality (also referred to as mixed reality), which blends real and virtual information in an attempt to optimize performance. Shapira et al. (2016) compared traditional and tactile virtual reality and found that when using virtual reality, social engagement among children was initially poor, after moving some of the game elements from the virtual to tactile, interactions among children increased and measures of social engagement improved. Further, Shapira et al. (2016) report that children in the study learned complex tasks faster in tactile virtual reality than in traditional virtual reality. Taken together, the results of these studies support the idea that the hMNS system is involved in communication, and that it is likely tuned to a specific set of parameters that evolved over millions of years of live social interactions. Disruptions in spatial and, importantly, temporal parameters may not have clear behavioral implications, but emergent neuroimaging of hMNS during virtual or robotic observations of actions suggest that there may be compensatory processing to resolve the changes in information. This compensatory processing in some cases means recruiting a broader network of areas or differences in the magnitude of activation in regions traditionally thought to be involved in social communication.

## Summary and Conclusion

The disruptive effect of spatial and– in particular– temporal discontinuity or asynchrony is not limited to digital communication, but extends generally to integration of multisensory events (Spence and Squire, 2003; Van Atteveldt et al., 2007; Sella et al., 2014; see Lewkowicz, 2000 for developmental view). In one study, Sella et al. (2014) recorded ERP latencies in an interactive, but non-social, virtual reality (VR) game setting. They found that the timing of adults’ cortical multisensory integration of events corresponded to the complexity of the signal (uni-, bi-, or tri-modal), such that the more complex the signal, the shorter the latency. Furthermore, reducing latency led to improvements in behavioral performance. Future research investigating multisensory events in a social VR context is needed to disentangle the relationship between the functioning of the hMNS and the impact of changes in spatial, temporal, and social information on the efficacy of multisensory integration. It is likely that multisensory integration is critically involved in communication and effective integration may mediate hMNS function. With respect to the role of digital technologies and the hMNS, the asynchronies (e.g., lack of contingency and congruency) inherent to virtual, screen-based communications (see Derks et al., 2008), such as video conferencing/video chat, email, and instant messaging, are likely disruptive and contribute to inaccurate or incomplete perceptions, compared to synchronous live interactions. The hMNS supports binding or creating correspondence between the visual information produced by an actor and the motor system of an observer. Indeed, the effectiveness and efficiency of this system may hinge on the fine-tuned binding windows characteristic of multisensory integration processing, and the flexibility of this system to adapt to the larger windows introduced by virtual correspondence is unknown. While there is a wealth of literature establishing this link and its importance in communication, little work has been done to establish the temporal and spatial integration windows that optimally support the sensorimotor binding critical for communication. Understanding the integration windows critical for communication has important implications for the continued use and development of screen-based and virtual communications. Further, this type of work would provide a strong demonstration of the functional role of the hMNS in social learning and communication. Advances in virtual and mixed reality as well as robotics will be key in teasing apart the extent to which disruptions can be accounted for by multisensory integration processes and by hMNS processes.

There are still many unanswered questions related to social learning, communication and the mechanisms underlying these functions as they relate to screen media and virtual social interactions. In order to extend the mirror neuron hypothesis to modern and increasingly virtual social engagement, researchers must begin to focus on addressing how low-level perceptual changes such as spatial discontinuity (e.g., differences in size, scale and resolution) and temporal asynchrony may influence the perceived contingency, and ultimately, perceived validity and utility of an interaction.

## Author Contributions

KD is the primary author of this work. KD determined the scope and focus of the paper and was the primary generator of all of the written content. KD worked with PG and AM to develop the central thesis of the paper. PG is the mentor and graduate advisor to KD and AM. He provided guidance during the formation of the central thesis of the review as well as detailed editing and commenting of each iteration of the draft. AM is a graduate student in PG’s lab and has a broad background in human imitation and the neural correlates thereof. AM provided critical insights that guided the shaping of the sections on anatomy and physiology. KD, PG, and AM contributed equally to the revised version of the manuscript.

## Conflict of Interest Statement

The authors declare that the research was conducted in the absence of any commercial or financial relationships that could be construed as a potential conflict of interest.
